# Non-targeted LC–MS/MS metabolomic profiling of human plasma uncovers a novel Mediterranean diet biomarker panel

**DOI:** 10.1007/s11306-023-02058-y

**Published:** 2023-12-08

**Authors:** Shirin Macias, Ali Yilmaz, Joseph Kirma, Sarah E. Moore, Jayne V. Woodside, Stewart F. Graham, Brian D. Green

**Affiliations:** 1 Institute for Global Food Security, School of Biological Sciences, Queen’s University Belfast, Belfast, UK; 2 Metabolomics Department, Corewell Health Research Institute, 3811 W. 13 Mile Road, Royal Oak, MI 48073 USA; 3 Michigan Medicine University of Michigan, Ann Arbor, MI 48109 USA; 4 Centre for Public Health, Queen’s University Belfast, Belfast, BT12 6BA UK; 5 Department of Obstetrics and Gynaecology, Corewell Health William Beaumont University Hospital, 3601 W.13 Mile Road, Royal Oak, MI 48073 USA

**Keywords:** Biomarkers, Mediterranean diet, Metabolomics, Dietary patterns, Mass spectrometry

## Abstract

**Introduction:**

Consumption of a Mediterranean diet (MD) has established health benefits, and the identification of novel biomarkers could enable objective monitoring of dietary pattern adherence.

**Objectives:**

The present investigation performed untargeted metabolomics on blood plasma from a controlled study of MD adherence, to identify novel blood-based metabolite biomarkers associated with the MD pattern, and to build a logistic regression model that could be used to characterise MD adherence.

**Methods:**

A hundred and thirty-five plasma samples from n = 58 patients collected at different time points were available. Using a 14-point scale MD Score (MDS) subjects were divided into ‘high’ or ‘low’ MDS adherence groups and liquid chromatography-mass spectrometry (LC–MS/MS) was applied for analysis.

**Results:**

The strongest association with MDS was pectenotoxin 2 seco acid (r = 0.53; ROC = 0.78), a non-toxic marine xenobiotic metabolite. Several lipids were useful biomarkers including eicosapentaenoic acid, the structurally related lysophospholipid (20:5(5Z,8Z,11Z,14Z,17Z)/0:0), a phosphatidylcholine (P-18:1(9Z)/16:0) and also xi-8-hydroxyhexadecanedioic acid. Two metabolites negatively correlated with MDS, these were the monoacylglycerides (0:0/16:1(9Z)/0:0) and (0:0/20:3(5Z,8Z,11Z)/0:0). By stepwise elimination we selected a panel of 3 highly discriminatory metabolites and developed a linear regression model which identified ‘high MDS’ individuals with high sensitivity and specificity [AUC (95% CI) 0.83 (0.76–0.97)].

**Conclusion:**

Our study highlights the utility of metabolomics as an approach for developing novel panels of dietary biomarkers. Quantitative profiling of these metabolites is required to validate their utility for evaluating dietary adherence.

**Supplementary Information:**

The online version contains supplementary material available at 10.1007/s11306-023-02058-y.

## Introduction

In recent years, there has been a surge of interest in the discovery of dietary biomarkers, particularly markers of healthy and sustainable dietary patterns. This area has seen an expansion in the application of metabolite-profiling (or metabolomics) technologies such as liquid chromatography-mass spectrometry (LC–MS) and nuclear magnetic resonance (NMR) spectroscopy. Studies have profiled biofluids such as urine, blood or saliva, and used this data to distinguish individual participant’s adherence to patterns such as the Average Danish Diet (ADD) and the New Nordic Diet (NND) (Khakimov et al., 2016), the Baltic Sea diet (Playdon et al., 2017), and, most commonly, the MD (Fitó et al., 2016).

The MD is characterised by high consumption of fruit and vegetables, fish, legumes, nuts and olive oil; a moderate intake of wine, red meat and dairy, and low consumption of sweets and processed foods (Estruch et al., 2018; Martínez-González et al., 2012). Measurement of adherence to the MD involves the use of various Mediterranean diet scores (MDS) which are based on food groups considered important within a MD. Two examples of these are the 9-item MedDiet score (Martínez-González et al., 2004) and the *Prevención con Dieta Mediterránea* (PREDIMED) 14-item MedDiet score. A previous investigation in our group using ^1^H-NMR metabolomic profiling was employed to identify a number of blood plasma biomarkers for the MD. This uncovered several modestly discriminatory metabolites including citric acid, pyruvic acid, betaine, mannose, and myo-inositol (Macias et al., 2019). Three similar studies have also performed ^1^H-NMR profiling, although each of these studies examined urine rather than blood. One study found that MD adherence was associated with changes in the levels of 3-hydroxybutyrate, citric acid, and *cis*-aconitate, oleic acid, suberic acid, various amino acids and some microbial co-metabolites (Vázquez-Fresno et al., 2015). Another study identified 34 metabolites associated with low or high adherence to MD (Almanza-Aguilera et al., 2017). A third NMR study found higher urinary hippurate in individuals consuming a MD (supplemented with Coenzyme Q10) compared with individuals consuming a Western diet rich in saturated fat (González-Guardia et al., 2015). There are some disadvantages with ^1^H-NMR metabolomic profiling, firstly that relatively few metabolites are measured and, secondly, that it lacks the sensitivity of other analytical platforms such as LC–MS (Brennan, 2014).

We are only aware of two studies that have applied untargeted LC–MS-based metabolomics in the search for blood biomarkers of a MD dietary pattern. Both studies found a major role of lipids, in particular, lysophospholipids to be discriminant metabolites of MD adherence. The first compared four different diet quality indices in male Finish smokers and correlated them with serum metabolites: the Healthy Eating Index (HEI), the Alternate Mediterranean Diet Score (aMED), the WHO Healthy Diet Indicator (HDI), and the Baltic Sea Diet (BSD). A total of 46 metabolites were associated with a MD pattern measured by aMED, 21 of which were identifiable, including 4 amino acids, 1 carbohydrate, 2 vitamins/cofactors, 11 lipids and 3 xenobiotics. Moderate and low correlations were observed between these metabolites and aMED, with the highest correlation corresponding to the lipid 1-myristoleoylglycerophosphocholine (14:1) (r = − 0.3). Metabolic pathway analysis showed 9 pathways were associated with higher aMED scores, among them the lysolipid pathway and the essential PUFA (Playdon et al., 2017).The second reported study was the Reduction of the Metabolic Syndrome in Navarra-Spain (RESMENA) intervention study involved 72 participants with high body mass index (BMI) and at least two features of Metabolic Syndrome (Bondia-Pons et al., 2015). Participants followed either a Mediterranean Diet or a control diet in accordance with American Heart Association guidelines for a period of 6 months (2 months active intervention and 4 months self-administered). Results showed that levels of several blood lipids (mainly lipids and lysophospholipids) were altered by a MD-based intervention after 2 months. Notably the phosphatidylcholine PC (P-18:1/20:3) was significantly higher in the MD group (p = 1E10 − 4) and the most discriminative metabolite between the 2 diets, accompanied by LysoPC (20:5) (p = 0.001), EPA (p = 0.004) and LysoPC (22:6) (p = 0.009),however these changes decreased from the second to the sixth month of the study, possibly due to lack of adherence to a MD during the self-adherence months (Bondia-Pons et al., 2015) A significant decrease in palmitic acid plasma levels was observed at the end of the 6 months intervention compared to baseline (p = 0.023).

The overall aim of the current study was to discover novel blood-based metabolite biomarkers associated with the MD pattern. We performed high LC–MS metabolomic analysis on blood plasma from a controlled study of MD adherence in a Northern European population. We then attempted to identify those ions with strong biomarker performance and to select an optimised biomarker panel. Lastly, using the identified metabolites, we built a logistic regression model that could best reflect MD adherence.

## Materials and methods

The Mediterranean Diet in Northern Ireland (MEDDINI) study was a pilot randomised controlled parallel group trial where 61 patients previously diagnosed with coronary heart disease (CHD) were recruited (135 plasma samples from n = 58 patients collected at different time points were available for the present study) from the Cardiology Directorate, Royal Victoria Hospital, Belfast. Patients provided informed written consent and were aged between 39 and 78 years. Seven-day food diaries were used to collect food consumption data. Patients were asked to record the foods consumed over seven consecutive days, including an estimation of quantity consumed and information on preparation methods used. From seven-day food diaries, a database was created registering all food amounts eaten by all patients during the course of the intervention (baseline, 6 months, and 12 months). Foods portions were described in detail in the food diaries and all amounts were registered in grams/day in the database. Further details from the intervention study have been previously described and reported elsewhere (Logan et al., 2010; Macias et al., 2019).

Food diaries from patients were scored using the validated 14-point MDS questionnaire based on the PREDIMED score. Scores are based on the answers to 14 questions along with food diaries. The questionnaire considered both the type of food and its frequency of intake (Supplementary information Table S1). A score of 0 indicated lowest adherence to an MD and a score of 14 indicated highest adherence. Recent MD advice was taken into consideration and types and quantities of foods within the PREDIMED score were adapted to reflect the typical diet and dietary recommendations in Northern Ireland. After scoring all patients food diets, the 14-point MDS was used to divide the samples into two groups. split by the median (Low and High MDS). Details have been previously described in Sects. 2.2.2 and 2.2.4 (Macias et al., 2019).

### Sample preparation

A total of 135 plasma samples from 58 participants from the MEDDINI study were analysed with the Dionex Ultimate 3000 UHPLC system coupled to an LTQ Orbitrap Elite mass spectrometer. This method was chosen for its high sensitivity. The extraction method was as follows: Plasma samples were stored at − 80 °C. Subsequently, all samples were thawed slowly on ice for 30 min prior to extraction, then 300 µL of ice cold methanol was added 100 µL of plasma, mixed for 10 min at 700 rpm, and subsequently centrifuged at 13,000×*g* for 15 min under vacuum, and reconstituted in 100 µL of ultra-pure water. Samples were then filtered by centrifugation using a 0.22 µm Costar spin-X centrifuge tube filter (8000×*g* at 4 °C for 5 min; Corning Incorporated, Corning, NY 14831, USA) and transferred to maximum recovery vials for analysis.

### UPLC-MS analysis

All solvents were purchased from Fisher Scientific (Pittsburg, USA) and were LC–MS grade or equivalent. Chromatography was performed on a Dionex Ultimate 3000 UHPLC system (Dionex, Softron GmbH, Germany) coupled to an LTQ Orbitrap Elite mass spectrometer (Thermo Fisher Scientific, Bremen, Germany). 5 µL of extracted plasma was injected (n = 3 injections per sample) onto an Acquity UPLC CSH C18 column (2.1 × 100 mm, 1.7 µm, Waters, Wexford, Ireland) operating at 50 °C and applying a binary mobile phase. The sample manager temperature was maintained at 4 °C and the order in which the samples were injected was randomised throughout the experiment. The gradient elution buffers were A (water with 0.1% formic acid (vol/vol)) and B (methanol with 0.1% formic acid (vol/vol)). Solvent B was varied as follows: 0 min 1%, 2.5 min 1%, 16 min 99%, 18 min 99%, 18.1 min 1% and 20 min 1% with a flow rate of 0.4 mL.min-1. Positive ionisation mode was employed with these conditions; source heater temperature at 400 °C, sheath gas at 60 arbitrary units (AU), aux gas at 45 (AU) and sweep gas at 1 (AU), capillary temp was maintained at 325 °C and source voltage at 3.5 kV. Mass spectra data were acquired in profile mode over the 50–1200 m/z range with a mass resolution of 60,000 at mass 400 (FWHM) and a scan time of 0.5 s. In further experiments, the samples were subjected to mass fragmentation analysis (FT HCD (10, 30 and 70 NCE), MS2) with an isolation width of 1 Da and 60,000 FWHM at 400 m/z. Prior to sample analysis 10 pooled conditioning samples were injected. To determine chromatographic reproducibility of retention times and peak intensities, pooled samples were injected after every 10 sample injections throughout the experiment (Cowan et al., 2016; Graham et al., 2013). Pooled samples were comprised of all plasma samples from the study and were subjected to the same extraction procedure applied to the individual plasma samples.

### Data analysis

UPLC-MS acquired data were analysed using Progenesis QI software (Waters Corporation, Milford, MA) for peak alignment, data normalisation and peak picking. Peak picking thresholds were set between 0.5 and 20 min. A peak threshold filter of 2.5 AU was applied (Cowan et al., 2016). Data was normalised to all compounds by correcting for multiple features to determine a global scaling factor. From 3548 features found on Progenesis, filtering was applied by selecting those ions with p-value < 0.01, fold change > 1.5 and the coefficient of variation (CV) was < 100%. Features with > 20%missing values were excluded. Filtered features were uploaded to Metaboanalyst where univariate (T-test) and multivariate (PCA, PLS-DA and O-PLS-DA) were performed and the model validated. Variable importance in projection (VIP) plots was used to identify the most influential metabolites responsible for the observed separation between groups in the PLS-DA model. Shortlisted features were ranked by their effect size. The effect size was calculated using the Effect size (Cohen’s d) to assess the impact of metabolite variability between the low and high MDS groups.Putative identifications were further examined to increase confidence in identification by mass fragmentation analysis whereby ms/ms spectra were used to search spectral libraries via additional online databases Metlin, HMDB (Wishart et al., 2009) and FooDB (2021).

### Metabolite identification

For each of the features related to the MDS obtained from Progenesis, identification was accomplished based on accurate mass (mass tolerance ≤ 4 ppm) and m/z values as matched against online databases. Adducts suggested by Progenesis were also matched with putative identifications from online libraries confirming that both neutral mass and m/z matched online databases. The confidence level of annotation was categorized according to the Metabolomics Standard Initiative (MSI) (Sumner et al., 2007). Fragment masses observed from MS/MS experiments were also searched for using Xcalibur™ Software—Thermo Fisher Scientific. For a further level of confidence, ion intensities were correlated to previously measured targeted biomarkers using the software IBM® SPSS® Statistics. The identified metabolites were further used for performing a logistic regression analysis that could maximise the AUC (ROC) of a panel of biomarkers using the software Metaboanalyst 4.0.

### Correlation with MDS and food groups

The association between putatively identified markers and MDS was analysed by correlating shortlisted ions of interest with the MDS. LC–MS data were non-normally distributed and hence metabolite correlations were examined using non-parametric correlations and Spearman’s rank correlation coefficient using SPSS.

From the criteria used to measure adherence to MD, 14 food groups were selected, and their values were correlated to the shortlisted ions of interest. These food groups were: fruit, fruit juice, vegetables, combined fruit with fruit juice and vegetables, fish, nuts, legumes, red meat, processed meat, chicken and turkey, whole grain cereals, alcohol beverages and sweet foods. Non-parametric correlations (Spearman r) were selected, and correlations were carried out using SPSS. All *p*-values underwent Benjamini–Hochberg correction (*q*-values) and were deemed significant if *p* ≤ 0.05 and *q* ≤ 0.05.

Previous targeted biomarker analysis was carried out with MEDDINI serum and plasma samples using HPLC and GC–MS (Logan et al., 2010). These were Vitamin C, EPA and TG. To obtain further confirmation, metabolites putatively identified were also correlated to targeted data using SPSS.

## Results

### Univariate analysis and identification of metabolites

A total of 135 plasma samples from 58 participants whose adherence to MD was assessed and scored over time (baseline, 6 months, and 12 months), were divided into either ‘low’ and ‘high’ MDS by splitting at the median. The highest MDS achieved by any individual on the 14-point scale was 10; hence, the resulting groups were: Low Score: (MDS: 0–4) (n = 63) and High Score (MDS: 5–10) (n = 72).

A total of 3548 features were detected using Progenesis. After data filtering a total of 73 statistically significant features differed between low and high MDS groups. From the 73 ions, univariate analysis was applied to shortlist the most significant features.

Comprehensive analysis of fragmentation patterns was hindered by time and budget constraints, and instrument availability. Consequently, we classified the level of identification according to the Metabolomics Standard Initiative (MSI) guidelines, as level 2. This classification represents a putative annotation (identification) of compounds based on their correspondence with MS data from established databases and the existing literature. For the identification process, the databases used were HMDB, Metlin, and FoodDB. It was possible to assign metabolite identities to 7 features (ions of interest) from this list which corresponded with the top most significant ones. These were: two monoacylglyceride: MG (0:0/16:1(9Z)/0:0 and MG (0:0/20:3(5Z,8Z,11Z)/0:0)), four fatty acid metabolites (EPA, lysoPC (20:5(5Z,8Z,11Z,14Z,17Z)/0:0), PC(P-18:1(9Z)/16:0), and xi-8-hydroxyhexa- decanedioic acid), and one xenobiotic (Pectenotoxin-2 secoacid (PTX2SA)). These metaboliteswere ranked according to their effect sizes (Table 1): The characteristics and performance [effect size, p-value, FDR, and area under the ROC curve (AUC)] of each of these is outlined in detail in Table 1. The two monoacylglycerides were significantly higher in the ‘low’ MDS group. The other 5 metabolites were significantly higher in the ‘high’ MDS group.

**Table 1:** Characteristics of seven putatively identified metabolites with biomarker potential for the Mediterranean dietary pattern

	m/z	RT (min)	Low MDS (mean intensity)	Std. Dev	High MDS (mean intensity)	Std. Dev	Effect size (d)	p-value	FDR	ROC (AUC)	Charge	Adducts	Polarity	Putative identification	Tolerance	Chemical formula	Identifier(d)
1	894.5209	17.40	990.39	654.44	2142.43	1436.66	1.03	1.81E-8	4.76E-7	0.78	1	M + NH4	Positive	Pectenotoxin 2 secoacid	Δppm = 0	C_47_H_72_O_15_	HMDB0040133
2	766.5722	17.86	6762.24	4066.45	16134.27	15842.27	0.83	1.86E-7	2.10E-6	0.79	1	M + Na	Positive	PC(P-18:1(9Z)/16:0)	Δppm = 0	C_42_H_82_NO_7_P	HMDB07997
3	328.2602	15.57	27708.71	36777.19	6146.53	3961.89	− 0.82	2.37E-7	2.12E-6	0.74	1	M + Na	Positive	MG(0:0/16:1(9Z)/0:0)	Δppm = 4	C_19_H_36_O_4_	HMDB0011534
4	403.2801	15.99	3326.77	3522.58	1296.85	563.90	− 0.80	5.11E-6	1.83E-5	0.71	1	M + Na	Positive	MG(0:0/20:3(5Z,8Z,11Z)/0:0)	Δppm = 4	C_23_H_40_O_4_	HMDB0011545
5	541.3148	14.83	2876.45	1629.01	4922.09	3471.75	0.75	1.38E-8	4.76E-7	0.76	1	M + H	Positive	LysoPC(20:5(5Z,8Z,11Z,14Z,17Z)/0:0)	Δppm = 3	C_28_H_48_NO_7_P	HMDB0010397
6	302.2235	15.74	8142.76	3775.01	13893.73	11077.42	0.69	5.31E-8	1.04E-6	0.77	1	M + Na	Positive	Eicosapentaenoic acid	Δppm = 3	C_20_H_30_O_2_	HMDB0001999
7	302.2315	10.22	1870.19	1580.56	3383.72	2983,85	0.63	6.27E-5	1.83E-4	0.73	1	M + NH_4_-H_2_O	Positive	xi-8-Hydroxyhexadecanedioic acid	Δppm = 2	C_16_H_30_O_5_	HMDB37831

Table shows the univariate statistical analysis of plasma metabolites in patients from Low (0–4) (n = 63) and High (5–10) (n = 72) MDS groups. Average intensities of ranked metabolites in both Low and High MDS groups and their respective standard deviations (SD) are shown for ions (m/z) with retention times (RT), along with p-values, false discovery rate (FDR) corrected p-values, receiver operating characteristic (ROC) curve area under the curve (AUC), charge, adducts, polarity, mass tolerance, putative identity and ion neutral mass, putative identifications and proposed chemical formula

### Multivariate analysis

**Fig. 1:**
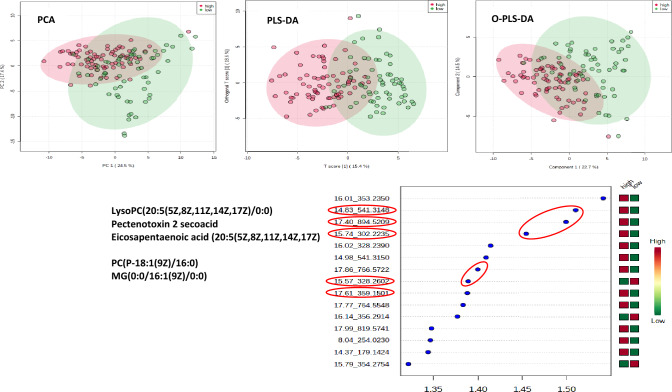
Multivariate statistical modelling of LC–MS data. Plots (top left, centre and right) shows group separations achieved by principal component analysis (PCA), orthogonal partial least squares discriminant analysis (OPLS-DA) and partial least squares discriminant analysis (PLS-DA), respectively. Red circles (1) represent patients with high MDS (5–10) and green circles (2) represent individuals with low MDS (0–4). Plot at the bottom is the resulting variable importance in projection (VIP) plot indicating the 15 most influential metabolites responsible for the observed separation in the PLS-DA model

Figure 1 shows principal component analysis (PCA) of the 73 ions of the LC–MS data. Principal component 1 (PC1) explained 24.5% of the variance and component 2 (PC2) explained 17.4% of the variance. Two supervised multivariate methods were then applied; PLS-DA, and orthogonal partial least squares discriminant analysis (O-PLS-DA) both improved the separation between the groups. The PLS-DA model was subsequently cross-validated on Metaboanalyst using the tenfold cross validation method (Supplementary Figure S1). The validation of the model showed R2 of 0.66 and Q2 of 0.48 after using a maximum of 4 components. Variable importance in projection (VIP) scores corroborated the univariate results, revealing that out of the 7 shortlisted metabolites, 5 ranked among the top 15 most influential metabolites responsible for the observed separation in the PLS-DA model (Fig. 1).

### Correlations with MDS and food groups

Correlations with MDS were also determined for each of 7 metabolites as well as food group-metabolite correlations (Table 2). The 2 monoacylglycerides (MG): MG (0:0/16:1(9Z)/0:0), MG (0:0/20:3(5Z,8Z11Z)/0:0) were significantly (p < 0.05) negatively correlated with MDS. The other 5 metabolites: EPA (ion), LysoPC (20:5(5Z,8Z,11Z,14Z,17Z)/0:0), PTX2SA, PC (P-18:1(9Z)/16:0), and xi-8-Hidroxyhexa decanedioic acid were significantly positively correlated with MDS (Table 2).

**Table 2:** Correlation of metabolites with MDA and food intake

Consumption of food grouping (g/day)		MG (0:0/20:3(5Z,8Z,11Z)/0:0)	EPA	LysoPC (20:5(5Z,8Z,11Z,14Z,17Z)/0:0)	PC(P-18:1(9Z)/16:0)	MG (0:0/16:1(9Z)/0:0)	Pectenotoxin 2 secoacid	xi-8-Hydroxyhexadecanedioic acid
MDS	r	− 0.405	0.352	0.432	0.511	− 0.464	0.534	0.444
	p	**1.0E-6**	**6.9E-5**	**1.6E-7**	**1.04E-9**	**1.40E-8**	**2.6E-11**	**6.83E-8**
	q	**1.0E-5**	**3.0E-4**	**1.9E-6**	**3.9E-8**	**2.8E-7**	**3.1E-9**	**1.0E-6**
Fruit and fruit juice	r	− 0.330	0.197	0.238	0.362	− 0.381	0.360	0.387
	p	**9.3E-5**	**0.023**	**0.005**	**1.6E-5**	**5.0E-6**	**1.8E-5**	**5.0E-6**
	q	**3.8E-4**	**0.042**	**0.010**	**1.0E-4**	**3.7E-5**	**1.0E-4**	**3.7E-5**
Fruit	r	− 0.280	0.188	0.181	0.310	− 0.354	0.328	0.359
	p	**0.001**	**0.030**	**0.035**	**2.5E-4**	**2.6E-5**	**1.1E-4**	**2.4E-5**
	q	**0.002**	0.052	0.058	**8.1E-4**	**1.3E-4**	**4.2E-4**	**1.2E-4**
Vegetables	r	− 0.147	0.183	0.197	0.133	− 0.080	0.186	0.192
	p	0.092	**0.036**	**0.024**	0.129	0.356	**0.033**	**0.027**
	q	0.134	0.059	**0.043**	0.173	0.423	0.055	**0.047**
Fruit, fruit juice and vegetables	r	− 0.319	0.230	0.293	0.350	− 0.356	0.378	0.401
	p	**1.6E-4**	**0.008**	**0.001**	**3.2E-5**	**2.2E-5**	**6.0E-6**	**2.0E-6**
	q	**6.0E-4**	**0.016**	**0.025**	**1.5E-4**	**1.2E-4**	**4.2E-5**	**1.84E-5**
Legumes	r	− 0.074	− 0.051	− 0.046	0.070	− 0.084	− 0.025	− 0.113
	p	0.399	0.558	0.599	0.428	0.336	0.776	0.198
	q	0.464	0.625	0.659	0.493	0.403	0.809	0.252
Fish	r	− 0.204	0.426	0.524	0.492	− 0.315	0.459	0.318
	p	**0.019**	**2.5E-7**	**7.2E-11**	**1.3E-9**	**1.9E-4**	**3.1E-8**	**2.0E-4**
	q	**0.036**	**2.72E-6**	**4.32E-9**	**3.9E-8**	**6.7E-4**	**5.31E-7**	**6.8E-4**
Red meat	r	0.078	− 0.177	− 0.133	− 0.206	0.151	− 0.258	− 0.237
	p	0.373	**0.042**	0.129	**0.018**	0.084	**0.003**	**0.006**
	q	0.438	0.067	0.173	**0.034**	0.124	**6.6E-3**	**0.012**
Processed meat	r	0.437	− 0.307	− 0.316	− 0.383	0.294	− 0.364	− 0.349
	p	**1.1E-7**	**2.9E-4**	**1.8E-4**	**5.0E-6**	**5.3E-4**	**1.8E-5**	**4.1E-5**
	q	**1.4E-6**	**8.9E-4**	**6.5E-4**	**3.7E-5**	**1.5E-3**	**1.0E-4**	**1.8E-4**
White meat	r	− 0.165	0.014	0.015	− 0.021	− 0.094	0.137	0.163
	p	0.059	0.875	0.867	0.814	0.283	0.118	0.061
	q	0.093	0.882	0.881	0.842	0.343	0.166	0.095
Cereals	r	− 0.312	0.140	0.228	0.271	− 0.289	0.290	0.475
	p	**2.3E-4**	0.109	**0.009**	**.002**	**6.7E-4**	**0.001**	**8.5E-9**
	q	**7.6E-4**	0.155	**0.018**	**0.005**	**0.002**	**0.003**	**2.0E-7**
Sweets and carbonated drinks	r	0.198	− 0.041	− 0.097	− 0.062	0.113	− 0.192	− 0.152
	p	**0.023**	0.642	0.267	0.477	0.197	**0.027**	**0.082**
	q	**0.042**	0.688	0.330	0.545	0.252	**0.047**	0.123
Sweets	r	0.161	0.010	− 0.043	− 0.051	0.112	− 0.117	− 0.097
	p	0.066	0.908	0.628	0.565	0.203	0.183	0.270
	q	0.100	0.908	0.685	0.627	0.252	0.238	0.330
Alcohol	r	− 0.127	0.257	0.254	0.133	− 0.041	0.186	− 0.035
	p	0.145	**0.003**	**0.003**	0.129	0.643	**0.033**	0.688
	q	0.193	**0.006**	**0.006**	0.173	0.688	0.055	0.730
Nuts	r	− 0.126	0.281	0.264	0.265	− 0.133	0.307	0.162
	p	0.151	**9.4E-4**	**0.002**	**0.002**	0.128	**3.3E-4**	0.064
	q	0.199	**0.002**	**0.005**	**0.005**	0.173	**9.6E-4**	0.098

Table shows the Spearman rank correlations of shortlisted metabolites with food groups used in calculating MDS. Significant correlations (p < 0.05) are marked in bold. Benjamini–Hochberg multiple comparison correction was significant if (q < 0.05). Number of correlations (n = 120)

### Logistic regression model using identified metabolites

**Fig. 2:**
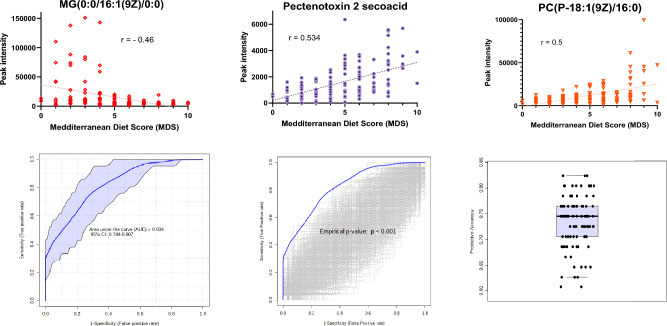
Optimised biomarker performance using logistic regression. Figure shows three individual plasma biomarkers: MG(0:0/16:1(9Z)/0:0), Pectenotoxin 2 secoacid and PC(P-18:1(9Z)/16:0), which were optimised as panel of plasma biomarkers with the resulting logistic regression curve. The correlation of each biomarker is shown against MDS with Spearman r values shown. Also shown is the resulting ROC curve (AUC = 0.834) with 95% CI (0.744–907). Also shown is the outcome of 1000 permutation tests, each of which re-assigns the Low/High MDS labels randomly to each sample and performs random sub-sampling cross-validation. None of the 1000 permutation tests were better than the original with the empirical p-value reported as p < 0.001

We then performed a range of logistic regression analyses based on combinations of the 7 putatively identified metabolites until optimised to obtain the highest possible ROC (AUC) value (Fig. 2). Using the intensities of MG(0:0/16:1(9Z)/0:0), PTX2SA, and PC(P-18:1(9Z)/16:0) and following cross validation (1000 permutations), we observed significant separation (p < 0.001) between Low and High MDS. We developed a logistic regression algorithm with an AUC (95% CI) 0.830 (0.763–0.894) with corresponding sensitivity and specificity equal to 0.794 (0.794–0.894)and 0.722 (0.619–0.826) respectively, following tenfold cross validation. Supplementary Table S2 lists the summary of each feature used to develop the following predictive algorithm:

$$\begin{aligned} {\text{logit}}\left( {\text{P}} \right) = & {\text{log}}\left( {{\text{P}}/\left( {1 - {\text{P}}} \right)} \right) = 0.313 \\ & - {\text{MG}}\left( {0:0/16:1\left( {9{\text{Z}}} \right)/0:0} \right) \\ & - 0.001{\text{Pectenotoxin}} - 2{\text{secoacid}} \\ & - {\text{PC}}\left( {{\text{P}} - 18:1\left( {9{\text{Z}}} \right)/16:0} \right) \\ \end{aligned}$$

where P is Pr(y = 1|x). The best threshold (or Cutoff) for the predicted P is 0.44

## Discussion

The present study acquired food diary data and concomitant blood samples from a Mediterranean diet intervention study in a Northern European population. Non-targeted metabolomic profiling was performed on plasma and this was combined with the calculated MDS to shortlist 73 features significantly differing between ‘low’ and ‘high’ consumers of a Mediterranean diet. Ultimately, this led to the putative identification of 7 high performing metabolite biomarkers (ROCAUC ≤ 0.79), which were also highly influential in multivariate modelling, and strongly correlated with MDS. Correlation of these biomarkers against each of the food groups involved in the calculation of MDS provided potential information on the possible origin of some of these. Using logistic regression analysis, we developed a model which accurately distinguished between the two dietary groups. We developed a predictive algorithm using the acquired data which had an AUC (95% CI) 0.83 (0.76–0.89) with corresponding sensitivity and specificity equal to 0.79 (0.79–0.89) and 0.72 (0.62–0.83). To our knowledge, no logistic regression model currently exists for human plasma that distinguishes low and high MDS with such a high degree of accuracy.

The results appear impressive, given that adherence in the MEDDINI intervention study (from which samples and data were obtained) would be described as sub-maximal for MD (Maximum MDS achieved was 10 from the 14-point scale. Mean MDS in the High MDS group was 6.68). Participants were not supplemented with any foods, and as such, did not eat identical foods. We should be mindful of the potential complexities here, particularly that the participants had a history of CVD and were taking prescribed medications. However, a number of the metabolites uncovered as biomarkers have obvious connections with food consumption (especially those with ROCAUC values > 0.7). Top performing putative biomarkers included PC (P-18:1(9Z)/16:0), EPA (an n-3 long chain polyunsaturated fatty acid), and a closely related metabolite LysoPC(20:5(5Z,8Z,11Z,14Z,17Z)/0:0). The strong correlation of each of these, not only with MDS, but also with fish consumption underscores dietary relevance. MEDDINI participants did not take any fish oil supplements, which rules this out as a possible source.

EPA has previously been reported as a validated biomarker of fish intake (Cuparencu et al., 2019), but the lysophospholipid metabolite performed substantially better than the free fatty acid (spearman r = 0.524 vs 0.426). This novel biomarker ought to be closely examined in future dietary biomarker studies, but it is encouraging that one other study is supportive of it as a biomarker of MD (Bondia-Pons et al., 2015). Unfortunately, due to time, budget, and instrument availability it was not possible to complete the MS–MS fragmentation analysis or to check metabolite identifications against analytical standards. However, an advantageous aspect of the present study was the correlation between intensity values of key metabolites, such as EPA and LysoPC(20:5(5Z,8Z,11Z,14Z,17Z)/0:0), with quantitated values from a previously published investigation. This prior study employed a targeted analysis of 31 biomarkers, quantifying EPA in the same plasma samples (Logan et al., 2010). Despite differences in units used for the two EPA compounds (AU vs. μM), strong associations were evident (r = 0.595; p = 6.9E-14 and r = 0.589; p = 1.3E-13, respectively), thus providing reasonable confidence in their identity. Fish oil supplementation studies in human volunteers indicate that LysoPC (20:5(5Z,8Z,11Z,14Z,17Z)/0:0) is associated with EPA intake (Block et al., 2010). It is thought that Lyso PC(20:5(5Z,8Z,11Z,14Z,17Z)/0:0) is more bioavailable than the free fatty acid, as gavage studies in mice increased the levels of EPA in the brain by > 100-fold (Yalagala et al., 2019).

Despite the potential utility of eicosapentaenoic acid metabolites, ultimately, none of these were incorporated into the optimised logistic regression model predicting MDS group. The three metabolites incorporated were MG(0:0/16:1(9Z)/0:0), PTX2SA, and PC(P-18:1(9Z)/16:0). The combined correlation of the 3 metabolites included in the model and MDS was (r = 0.64; p = 5.6E-17). MG(0:0/16:1(9Z)/0:0) was one of two monoacylglyceride biomarkers identified (the other being MG(0:0/20:3(5Z,8Z,11Z)/0:0). The respective fatty acid components of these MGs have potentially strong dietary relevance, and both markers significantly decreased with increasing MDS. Both of these metabolites were correlated with previous targeted measured blood triglycerides showing a highly significant association (r = 0.511; p = 1.2E-9) and (r = 0.348; p = 6.8E-5), respectively.

MG(0:0/20:3(5Z,8Z,11Z)/0:0) is the monoacylglyceride metabolite of mead acid (20:3(5Z,8Z,11Z). The levels of mead acid in plasma are known to be a marker for overall essential fatty acid (EFA) status (Fokkema et al., 2002; Mead acid, 2021). Mead acid is not itself considered an essential fatty acid, but in the absence of adequate essential fatty acids in human tissues, the fatty acid MG(0:0/20:3(5Z,8Z,11Z)/0:0) is metabolised to mead acid (Gramlich et al., 2019; Kinoshita et al., 2016; Mead acid, 2021; Strandvik, 2006). Given that MG(0:0/20:3(5Z,8Z,11Z)/0:0) is significantly negatively correlated with MDS potentially indicates that some individuals in the ‘low’ MDS group, particularly those with very high MG(0:0/20:3(5Z,8Z,11Z)/0:0) levels, may exhibit deficiency in essential fatty acids (Kaur et al., 2014; Kris-Etherton et al., 2001). Adherence to MD has proved an enhancement in essential fatty acid levels (Hagfors et al., 2005; Ristic-Medic et al., 2020). This is consistent with our findings which showed a significant difference in EFA concentrations between low and high MD adherence groups (p = 0.001) and an 11.10% increase between low and high MD adherence.

Alternatively, it is also possible that MG (0:0/20:3(5Z,8Z,11Z)/0:0) is derived from the diet. For instance, mead acid is present in very high levels in animal cartilage, and it is noteworthy that this metabolite is strongly correlated with processed meat intake. The other MG biomarker identified is a metabolite of trans-palmitoleic acid (16:1(9Z)) which has previously been established as a marker of full fat dairy intake (Pranger et al., 2019). Unfortunately, the correlation between this ion and dairy intake was not statistically significant in our study (p = 0.12).

Another novel high performing MDS biomarker which correlated strongly with fish consumption is a xenobiotic compound called Pectenotoxin-2 seco acid (PTX2SA). PTX2SA correlated closely with both MDS and fish intake. Produced by toxic dinoflagellates, pectenotoxins accumulate in shellfish, and humans can potentially be exposed to these through shellfish consumption. PTX2 is one of the family of pectenotoxin compounds, which are polyether macrolide toxins responsible for diarrheic shellfish poisoning (2021). Reassuringly however, PTX2sa is a non-toxic metabolite of PTX2. Injection of mice with doses as high as 5 mg/kg does not cause toxicity, and oral administration of PTX2sa is likely to be even less toxic (Miles et al., 2006). Intriguingly, PTX2sa and its epimer 7-epi-PTX2sa have previously been detected in Irish waters (Daiguji et al., 1998) however, we cannot find any evidence that PTX2sa has been detected in humans before. PTX2 is highly lipophilic and may not be released and absorbed during human digestion of shellfish. It also appears to be quite labile and, if it were to be liberated during digestion, acidity in the stomach would rapidly lead to metabolism to its non-toxic seco acid. PTX2sa has been detected in various marine samples, with mollusks and plankton being the most abundant sources (Vale & Sampayo, 2002). We are not aware that PTX2 or PTX2sa has been detected in fish specimens before, however, it seems likely given that trace amounts will occur, given that PTX2 has been measured at ≤ 8 ng/l in seawater, ≤ 10 ng in suspended particular matter and ≤ 2 ng/g in marine sediment (Chen et al., 2017). It is entirely plausible that this compound could originate from fish intake. Mollusk/shellfish consumption among MEDDINI participants during the surveyed period was, however, extremely rare, and the calculation of MDS was based on fish intake, and not shellfish intake. Alternatively, given the fact that PTX2/PTX2sa are highly lipophilic they may persist long after absorption and may reflect shellfish intake outside of the food diary data collection period.

The third metabolite to be incorporated in the model was a phosphatidylcholine identified PC(16:0/18:1(11Z)), comprised of palmitic acid and vaccenic acid. This metabolite was the best performing individual biomarker overall (AUCROC = 0.79) and it strongly positively correlated with MDS (r = 0.495; p = 1.04E − 9). It is difficult to pinpoint the dietary origin of PC(16:0/18:1(11Z)) as it correlated with a number of food types, but the strongest association was with fish intake (r = 0.492, p-value = 1.3E − 9)), which was almost equal in strength to its association with MDS (r = 0.51; p = 1.04E − 9). Interestingly, it is not the first time this metabolite has been associated to MD. The association of EPA and PC(P-18:1(9Z)/16:0)) with fish and MD adherence has been previously reported in the MD internvention study RESMENA, and it was reasuring to see our findings were consistent with those of RESMENA MD intervention study.

One other noteworthy MDS biomarker detected was Xi-8-Hydroxyhexadecanedioic acid (Xi-8-HHDDA), also known as 8-hydroxyhexadecane dioic acid. Xi-8-HHDDA is a long-chain fatty acid, and here it was significantly associated with fruit, fruit juice and vegetable intake, and it also correlated with blood plasma levels of vitamin C (r = 0.30; p = 8E − 5). It seems likely to originate from dietary plant intake, given that it is a cutin constituent of fruits and vegetables (ContaminantDB, 2021; Metabolome & Database, 2020). Its presence has been reported in fruit and tomatoes, which makes it a potential biomarker for the consumption of these food products (2021), Kosma et al., 2010). It has also been identified as one of the major constituents of sweet cherries (Peschel et al., 2007). Other dioic acids have been reported in other fruits, for example, 10,16-dihydroxyhexadecanoic was identified as major components of the of the cuticle of different apple varieties (Holloway, 1973). To the best of our knowledge, this is the first time that the metabolite xi-8-Hydroxyhexadecanedioic has been identified as a potential biomarker of fruits and vegetable intake.

The limitations of the present study should be taken into consideration for future studies. A total of 58 patients participated in the study followed an MD during 12 months and plasma samples were collected at baseline, 6 months and 12 months. Samples were divided into two groups (low and high MDS) according to how well patients adhere to the 14-point scale MDS. Despite the dependency of samples within groups, our multivariate analysis did not indicate that profiles from the same patient were more similar to each other than to other patients. Notably, most participants started with low MDS at baseline and improved during the intervention. The majority of samples in the ‘Low MDS’ group were from baseline (n = 56 out of 63), while most samples in the ‘High MDS’ group were from the 6 and 12 months time points (72 out of 79). Paired analysis was also conducted (timepoint-based) which showing the same top metabolites as the primary analysis. This observation underscores the dietary intervention as the predominant factor influencing participant profiles.

Additional investigation and identification of LC–MS biomarkers such as a comprehensive fragmentation analysis and comparison against analytical standards was unfortunately not possible due to time, budget and instrument availability. However, the intensity values for three metabolites were correlated against quantitated values acquired from a previously published investigation (Logan et al., 2010) which found strong associations thus providing reasonable confidence of their identity.During the intervention, participants were encouraged to make dietary changes to align with the MD, which included recommendations to replace alcohol with wine, reduce the use of butter and margarine, and incorporate olive oil, olive oil spreads, and rapeseed oil. However, it’s important to note that the actual intake of wine, nuts, and legumes did not significantly increase among the participants, with p-values of 0.54, 0.10, and 0.58, respectively. In contrast, the consumption of olive oil, olive oil spreads, and rapeseed oil did significantly increase (p < 0.001). Nevertheless, it’s worth mentioning that only 64.3% of participants included these items in their diets, and the quantities consumed were relatively low, with an average daily intake of 2 g.

The absence of markers associated with olive oil consumption in our study is not surprising, considering that these items are not typical components of the dietary habits of Northern European populations. It’s important to acknowledge that the maximum MD score achieved during the intervention was 10 on a 14-point MD scale. This limitation can be attributed to the significant differences in dietary preferences between non-Mediterranean and Mediterranean populations. However, it should be noted that our study identified biomarkers of fish and fruit intake and revealed reduced levels of putative biomarkers associated with processed foods, which are typical of MD adherence.

In conclusion, the present study is only the third to apply untargeted LC–MS metabolomics to a MD study and it provides a clear indication that this approach can be effective in a Northern European population with sub-maximal MD adherence. The findings further advance the ongoing search for a biomarker panel to determine adherence to a MD diet. Specifically, we propose a logistic regression model for accurately distinguishing low or high MDS, which will require careful validation using targeted and quantitative methods in other MD cohorts. There is clear evidence that the shortlisted metabolite biomarkers have statistically significant dietary associations (five for fish intake, one for fruit and vegetable intake, and two for processed meat), thus making the findings biologically plausible, and worthy of further investigation.

### Supplementary Information

Below is the link to the electronic supplementary material.

Supplementary file1 (DOCX 240 KB)

## Data Availability

Data uploaded to Metabolights: MTBLS7948: Non-targeted LC–MS/MS metabolomic profiling of human plasma uncovers a novel Mediterranean diet biomarker panel. https://www.ebi.ac.uk/metabolights/editor/console

## Code availability

The software used for data analysis in this study (Progenesis) is available via: https://www.nonlinear.com/progenesis/qi/ and the online software Metaboanalyst: https://www.metaboanalyst.ca/
