# Supplementary material for: Non-targeted LC–MS/MS metabolomic profiling of human plasma uncovers a novel Mediterranean diet biomarker panel
Source: Metabolomics. 2023 Dec 8;20(1):3. doi: 10.1007/s11306-023-02058-y (PMC10709258; doi:10.1007/s11306-023-02058-y)
Supplement: Supplementary file 1 — Supplementary file1 (DOCX 240 KB) [file 11306_2023_2058_MOESM1_ESM.docx]

##### **SUPPLEMENTARY INFORMATION**

##### *Table S1. Fourteen-point criteria used to measure adherence to MD on the MEDDINI study. FOURTEEN POINT MEDITERRANEAN DIET SCORE (MDS)*

| **Questions** | **Criteria for 1 point** |
| --- | --- |
| 1. Do you use olive oil or rapeseed oil as your main cooking fat? | Yes |
| 1. Do you use olive or rapeseed oil-based spreads (e.g. Golden olive, Olivio, Bertolli)? | Yes |
| 1. How much olive/ rapeseed oil do you consume in a given day? Including oil used for frying, salads, out-of-house meals etc (in tablespoons)   How much olive or rapeseed oil-based spread do you consume in a given day? (in teaspoons) | ≥4 tbsp oil / day  and/or  3 tsp spread/ day |
| 1. How many portions of fruit (including natural fruit juices) do you consume per day? (1 portion = 1 apple/banana (80g), small glass juice (150ml) | ≥2 portions/ day |
| 1. How many vegetable servings do you consume per day? Including raw/ cooked vegetables, salad but not including potatoes (1 serving: 3 tablespoons/80g) | ≥3 portions/ day |
| 1. How many servings of legumes (peas, beans and lentils including kidney beans, baked beans, chickpeas, red lentils etc) do you consume per week? (1 serving :3 tablespoons/ 80g) | ≥3 servings/ week |
| 1. How many servings of red meat including beef, pork, lamb and minced beef do you consume per week? (1 serving: medium portion/ 100–150 g) | ≤2 servings /week |
| 1. How many servings of processed meat including ham, bacon, sausages, meat pies and other meat products etc.) do you consume per week? (1 serving: medium portion/ 100–150 g) | ≤1 serving /week |
| 1. How many servings of chicken/ turkey do you consume per week? (1 serving: medium portion/ 100-150g) | 2 servings /week |
| 1. How many servings of fish (tuna, cod, haddock, salmon, mackerel, herring, and sardines etc, including tinned varieties, excluding crumbed or battered fish) or shellfish do you consume per week? (1 serving: 1 fillet/small fish or 140g) | ≥3 servings /week |
| 1. Do you preferentially consume wholegrain bread and/ or cereal and/ or rice and/ or pasta instead of non-wholegrain (white) varieties? | Yes |
| 1. How many servings of natural nuts do you consume per week? (1 serving: 1 small handful/ 30 g) | ≥3 servings /week |
| 1. How many times per week do you consume sweet foods (including biscuits, buns, pastries, chocolate, sweets, sweet or carbonated drinks or desserts)? | ≤3 times/ week |
| 1. How often would you consume up to 3 small glasses of wine or equivalent other alcoholic beverages per week? (1 small glass:125ml) | 1-3 glasses or equivalent ≥ 3 days/ week |

**
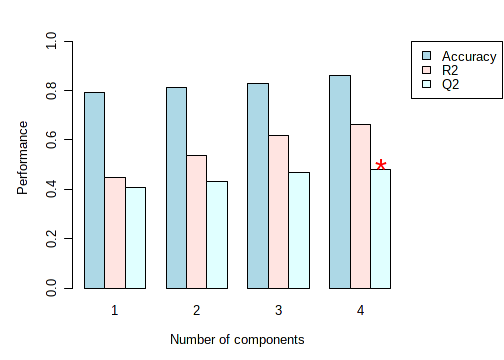
**

**
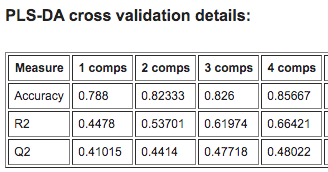
**

Figure S1. Cross validation of the PLS-DA multivariate model as performed using Metaboanalyst 4.0 (left) showing accuracy, R2 and Q2 model parameters. Validation based on 4 components (right)

Figure S2. Intensity values of putatively identified metabolite biomarkers of Mediterranean Diet Score (MDS)

Table S2. Summary of each feature used to develop the logistic regression predictive algorithm for Mediterranean Diet Score (MDS)

|  | **Estimate** | **Std. Error** | **z value** | **Pr(>\|z\|)** | **Odds** |
| --- | --- | --- | --- | --- | --- |
| **(Intercept)** | 0.313 | 0.555 | 0.564 | 0.573 | - |
| **MG(0:0/16:1(9Z)/0:0)**  **(15.57_328.2602)** | 0 | 0 | 2.724 | 0.006 | 1 |
| **Pectenotoxin 2 secoacid**  **(17.40_894.5209)** | -0.001 | 0 | -1.844 | 0.065 | 1 |
| **PC(P-18:1(9Z)/16:0)**  **(17.86_766.5722)** | 0 | 0 | -1.492 | 0.136 | 1 |
